# Binaural beats to entrain the brain? A systematic review of the effects of binaural beat stimulation on brain oscillatory activity, and the implications for psychological research and intervention

**DOI:** 10.1371/journal.pone.0286023

**Published:** 2023-05-19

**Authors:** Ruth Maria Ingendoh, Ella S. Posny, Angela Heine

**Affiliations:** Department of Psychology, University of Duisburg-Essen, Essen, Germany; La Sapienza University of Rome, ITALY

## Abstract

*Binaural beats* are an auditory phenomenon that occurs when two tones of different frequencies, which are presented separately to each ear, elicit the sensation of a third tone oscillating at the difference frequency of the two tones. Binaural beats can be perceived in the frequency range of about 1–30 Hz, a range that coincides with the main human EEG frequency bands. The *brainwave entrainment hypothesis*, which assumes that external stimulation at a certain frequency leads to the brain’s electrocortical activity oscillating at the same frequency, provides the basis for research on the effects of binaural beat stimulation on cognitive and affective states. Studies, particularly in more applied fields, usually refer to neuroscientific research demonstrating that binaural beats elicit systematic changes in EEG parameters. At first glance, however, the available literature on brainwave entrainment effects due to binaural beat stimulation appears to be inconclusive at best. The aim of the present systematic review is, thus, to synthesize existing empirical research. A sample of fourteen published studies met our criteria for inclusion. The results corroborate the impression of an overall inconsistency of empirical outcomes, with five studies reporting results in line with the brainwave entrainment hypothesis, eight studies reporting contradictory, and one mixed results. What is to be noticed is that the fourteen studies included in this review were very heterogeneous regarding the implementation of the binaural beats, the experimental designs, and the EEG parameters and analyses. The methodological heterogeneity in this field of study ultimately limits the comparability of research outcomes. The results of the present systematic review emphasize the need for standardization in study approaches so as to allow for reliable insight into brainwave entrainment effects in the future.

## Introduction

When people are presented with two acoustic signals of slightly different frequencies separately to each ear, the percept of a third tone oscillating at the difference of the two frequencies arises. The percept of this third tone is described as being located *in the head*, or *between the ears*. This psychoacoustic phenomenon is called the *binaural beat* [1]. When, for example, a tone with a frequency of 400 Hz is presented to the right ear and a second tone with a frequency of 420 Hz is presented to the left ear simultaneously, a binaural beat of 20 Hz will occur. The illusory nature of the binaural beat, a perceptual phenomenon that has no manifest external source, makes it of interest for research on sound perception and acoustic processing [2].

There are, however, a number of constraints to the perception of binaural beats. First, an early study by Licklider et al. [3] demonstrated that the two presented frequencies must be of a maximum of 1000 Hz to elicit a binaural beat. This is due to the fact that the human auditory pathway can only encode sound waves with frequencies up to 1 kHz [4]. Second, binaural beats seem to be best perceived at carrier frequencies—i.e. the frequencies of the two presented tones–of around 400 Hz [5]. Third, Perrott and Nelson [6] demonstrated that the maximum difference for the two tones must be around 30 Hz. Beyond frequency differences of 30 Hz, the two tones are perceived separately instead of eliciting the percept of a binaural beat. This threshold seems to vary depending on the stimulation technique, though [7].

The study by Licklider et al. [3] was the first to characterize the percept of the binaural beat depending on frequency differences in more detail. Up to 20 Hz, the binaural beat is described as a *tone fluctuating in loudness*, while frequency differences larger than 20 Hz elicit a *rough sound*. Below a difference of 3 Hz, the binaural beat is perceived as a *rotating tone*—a sound that appears to rotate in the head from ear to ear [8]. These thresholds are approximate and assumed to vary interindividually [2]. Furthermore, Schwarz and Taylor [4] found that participants can control the perception of binaural beats to a certain degree by focusing either on the two presented tones separately, or on the binaural beat percept instead. Differences between individuals in the ability to perceive binaural beats were also suggested [9].

Binaural beats are, as mentioned in the beginning, an *illusory* phenomenon, which means that its perceptual basis is not the interference of two sound waves, but a result of their combined neural activity elicited in the auditory pathway [10]. The *superior olivary complex* (SOC), the first locus of the auditory pathway to receive input from both ears and an important structure for sound integration [11], has been identified as the main neuroanatomical structure involved in binaural beat perception [12, 13]. The SOC is functionally implicated in the localization of sound in space. In addition to the processing of differences in loudness, sound localization is based on the identification of phase differences between the signals perceived by both ears [14]. Phase differences typically occur when the incoming sound signal is not fully centered but more lateralized toward one ear. The capacity of the SOC to process phase differences is essential for the binaural beat percept. For binaural beat stimulation, the tones are typically presented via headphones to guarantee that exposure to each of the two frequencies is restricted to one ear only [15]. This technique rules out the integration of external sounds, and, thus, allows for an interpretation of perceived frequency differences as phase differences [7].

A second type of auditory stimulation which is related to binaural beats, are *monaural beats* [16]. Beats are referred to as monaural when they are elicited by two tones of different frequencies presented to *one ear only*. So, in the case of monaural beats the actual interference of the sound waves is responsible for the beat percept, which makes it a phenomenon on the cochlear level [17]. Because of this, the monaural beat percept depends on similar intensity of the tones, whereas binaural beats can be perceived irrespective of differences in degree of loudness because their percept relies solely on phase differences [18]. While monaural beats are perceptible throughout the whole audible frequency range, binaural beats are a phenomenon of low frequencies [7]. This implies that monaural beats and binaural beats have quite different properties which, in turn, render them useful for different types of basic research and application.

Binaural beats, like monaural beats, fall into the range of *auditory modulated tones*, which are frequently used in basic acoustic research as well as in clinical diagnostics [19]. Any auditory stimulation using repetitive or modulated tones elicits a specific neural response pattern that can be measured by electroencephalography (EEG) or magnetoencephalography (MEG) [19]. Neural responses are detectable, for example, as *auditory steady state responses* (ASSRs) [20] or *auditory frequency-following responses* (FFRs), which both appear immediately after stimulus onset in the form of composite waves [21, 22]. ASSR and FFR can be functionally attributed to discrete stages of auditory processing and are, thus, both relevant for sound processing and hearing research [23]. Several studies demonstrated that stimulation with binaural beats elicits ASSRs as well as FFRs [1, 4, 13, 24].

Research on binaural beats started as early as 1839, when the phenomenon was first described by H. W. Dove [25]. However, given that it is a perceptual response to stimuli which are artificially generated and do not occur in natural settings, binaural beats were dismissed as a mere curiosity for more than a century. Scientific interest in binaural beats has been rekindled only much later when the results from early empirical studies were systematically integrated by Oster [9]. Not only did he describe the phenomenon in more detail, he also highlighted the potential relevance of binaural beat stimulation for practical use. This sparked a new wave of activity not only in the scientific community but also in certain pseudoscientific contexts. Ultimately, this led to the development of intervention approaches which use binaural beats for cognitive enhancement [26]. It was probably due to this latter trend, that basic scientific research on binaural beats was largely neglected in the decades following Oster’s publication [9]. It has been only for the last two decades that systematic research on binaural beats has gained traction again. This was mainly due to a series of studies in the 2000’s that investigated ASSRs using EEG and MEG, which provided first reliable neuroscientific evidence for specific responses in human brain activity after binaural beat stimulation [4, 13, 24]. The newly available empirical data on electrocortical responses to binaural beats led to a growing interest in the neuro-physiological correlates [27–29].

### The brainwave entrainment hypothesis

More recently, research has mainly focused on the psychological effects of binaural beat stimulation [30]. Within this field of study, effects on cognition, emotion, as well as certain concomitant physiological changes are investigated [31, 32]. The theoretical basis of psychological research on the effects of binaural beat stimulation is provided by the *brainwave entrainment hypothesis* [15] which suggests that auditory or visual stimulation at a specific frequency will lead the brain’s electrocortical activity to oscillate at the external signal’s frequency or at its multiples. For binaural beat stimulation, the brainwave entrainment hypothesis was corroborated primarily by empirical studies demonstrating time-locked ASSRs [4, 13, 24]. While review articles on BWE indicate that binaural beats are the most commonly used type of auditory stimulation, no specific justification is given for this preference [30].

What makes the possibility of brainwave entrainment (BWE) interesting for psychological research is that specific frequency bands of the human EEG are associated with different physiological and psychological states [33]. The commonly held assumption is that BWE may, thus, be a method to induce specific physiological and psychological states through stimulation within particular frequency bands [15]. Effects of binaural beat stimulation have, consequently, been investigated with respect to a variety of psychological phenomena associated with specific EEG frequency bands, such as aspects of cognitive processing [31, 34, 35], affective states [31, 36, 37], mood [1, 38, 39], pain perception [40, 41], meditation and relaxation [42, 43], mind wandering [44, 45], or creativity [46]. Research on changes in cognitive and affective states has also been carried out using monaural beats as an entrainment technique [47–49].

### The neuroscientific evidence for BWE

Given that the theoretical basis of the applied studies on effects of binaural beat stimulation is the brainwave entrainment hypothesis [15], the fact that a considerable number of available basic research studies failed to demonstrate sound evidence for BWE [36, 37, 40, 42, 44–46] makes the results of those applied research endeavors highly questionable. There are actually several studies yielding results that are difficult to bring in line with the assumption of brainwave entrainment. Using 7 Hz (EEG theta frequency) and 16 Hz (beta frequency) binaural beat stimulation, Goodin et al. [5] did not find any differences in average spectral power for the experimental condition compared to a white noise control condition. In the same vein, Gao et al. [50] did not find changes in relative power in response to five minutes of EEG delta, theta, alpha or beta binaural beat stimulation compared to a pink noise condition, and López-Caballero and Escera [51] were also not able to determine effects of binaural beat stimulation on spectral power in any of the major EEG frequency bands. Beyond that, there are a number of other studies that challenge the brainwave entrainment hypothesis [e.g., 25, 52–55]. The additional problem that in most studies on potential effects of binaural beat stimulation an entrainment was not independently empirically assured but merely *assumed*, has been pointed out before [40, 56].

In recent years, however, a number of neuroscientific studies provided at least partial evidence in favor of the brainwave entrainment hypothesis [e.g., 2, 57, 58]. For example, Schwarz and Taylor [4] found an ASSR with ten minutes of 40 Hz (gamma frequency) binaural beat stimulation in comparison to no-stimulation conditions. Draganova et al. [13] reported similar findings using MEG. Karino et al. [24] demonstrated BWE for stimulation in the delta (1- to 4-Hz range) and theta frequency bands. These latter findings were corroborated by Seifi Ala et al. [59], who applied 7 Hz binaural beat stimulation and found changes in relative theta power. Recently, Orozco Perez et al. [1] used continuous binaural beat stimulation at 7 Hz and 40 Hz and found both an ASSR in the respective beat frequency as well as an FFR in the carrier frequency (400 Hz). Research on monaural beat stimulation supports this inconclusiveness regarding the brainwave entrainment hypothesis, with some studies demonstrating BWE through monaural beat stimulation [16], while other did not find entrainment effects [47].

The conflicting findings regarding BWE can be attributed to a number of obvious problems in this field of research. First of all, the operationalization of entrainment effects in the human EEG is diverse, with some researchers considering the presence of time-locked responses in the auditory system (ASSR and FFR, respectively) as indicators of BWE [e.g., 1, 4, 52], while others focus on changes in EEG power measures (oscillatory activity) [5, 50, 51, 55]. Apart from studies looking into EEG measures in the frequency-domain, a number of studies link BWE to the time-domain (event-related potentials, ERPs) [57, 60, 61]. Adding even more heterogeneity to the measurement of BWE, recent studies have shifted their focus from effects in the EEG frequency- and time-domains to brain connectivity measures [1, 50, 59, 62–65].

A second limiting factor is that studies in the field tend to use highly heterogeneous study designs [52]. Heterogeneity starts with the study samples, with most studies involving healthy adult populations [e.g., 2, 51, 55, 57], while others examine neurological samples [16, 64]. Another problem is related to the variety of frequencies used for binaural beat stimulation. This is of special importance since it is assumed that different frequency bands differ in their capacity to be entrained [52]. Furthermore, there is considerable heterogeneity in the presentation of the binaural beats with some studies using tone bursts [e.g., 57], while others use continuous tones which further vary in their duration [e.g., 50]. In addition, there is considerable variation in overall study designs. While some studies used passive listening conditions with eyes open or eyes closed [e.g., 52, 59], in others participants were instructed to focus on the binaural beats, and still others aimed at distracting participants intentionally [e.g., 51, 62]. Finally, a number of study approaches had participants execute an unrelated additional task during binaural beat stimulation [e.g., 25, 65]. The available studies on the effects of binaural beats use a wide range of control conditions, that is pure tones, non-superimposed tones, monaural beats, or silence [e.g., 1, 25, 64]. A final issue is that when it comes to EEG and MEG measurement, studies differ considerably with respect to data collection and data processing procedures [e.g., 1, 61, 64], as well as data analysis [e.g., 54, 58, 61].

In view of this, the need for a systematic analysis and integration of the current state of research becomes evident—even more so in consideration of the fact that binaural beat stimulation is regularly used in applied contexts [1, 31, 34–46].

### The present systematic review

While research on potential psychological effects of binaural beat stimulation is based on the assumption of brainwave entrainment, the empirical basis for this presupposition is open to question given that neuroscientific results on BWE through binaural beat stimulation appear to be largely inconclusive. The aim of the present systematic review study is, thus, to provide an overview of brainwave entrainment effects in response to binaural beat stimulation. Hence, the research question is whether the available empirical evidence is sufficiently robust to assume that stimulation with binaural beats in the frequency range of the human EEG elicits systematic changes in EEG oscillatory activity in line with the brainwave entrainment hypothesis.

## Materials and methods

### Inclusion and exclusion criteria

This research question implies several criteria for the inclusion or exclusion of studies. To be included in the present systematic review, publications had to fulfill the following requirements: the studies (a) investigated neurologically healthy and normal-hearing adults, (b) implemented a binaural beat in the frequency range of the human EEG, (c) presented binaural beats with pure carrier tones in the human auditory range or pure audible tones embedded in noise, (d) included at least one control condition assumed not to elicit brainwave entrainment, and (e) investigated binaural beat perception in a passive state, which means that participants were not confronted with a secondary task during the intervention. The only exception from the latter requirement was when participants were allowed to watch a silent movie during the experimental session since this is common practice in hearing research to ensure implicit processing of auditory stimuli [66]. The implementation of a control condition as required by criterion (d) may have been realized either by comparing different experimental groups (between-subjects design), or by means of different experimental conditions compared within participants (within-subject design). Additional inclusion criteria were (f) explicit statements concerning the study aim to test for BWE, (g) the use of EEG, and (h) the report of at least one EEG measure to ascertain BWE. Since the scoping searches yielded grey literature as well as conference proceedings that were insufficient with respect to the information provided, another criterion for inclusion in this review was that (i) the studies had to be original, published research using an original dataset. Complementary to the inclusion criteria, the following exclusion criteria were applied: (a) the participants were under the age of 18 or suffered from neurological diseases or hearing disabilities, (b) the implemented frequencies for the intervention were not within the human EEG frequency range, (c) the binaural beats were not presented using pure carrier tones within the bounds of the human auditory range or were combined with other auditory or non-auditory stimuli, (d) the study did not include a control condition which was assumed to not elicit BWE or (e) included a secondary task during the intervention. Additional exclusion criteria were related to studies (f) not establishing BWE empirically, (g) not using EEG, (h) not consulting EEG correlates of BWE, or (i) not being published, original research with a dedicated dataset.

To systematically apply inclusion and exclusion criteria during the screening and selection process, a screening and selection tool was developed and used.

### Search

A systematic search was carried out on June 20, 2021, using *PubMed*, *Web of Science* (WoS) and *Scopus* as databases to identify available literature. The search was limited to English and German results. Publication time limits were not set. Scoping searches revealed that binaural beat research using EEG is so scarce that the broad search terms of *binaural beat(s)* and *EEG* were sufficiently specific. The databases were browsed for entries which contained the search terms in the title, abstract, or (if applicable) keywords. Details of the search in each of the databases as well as the syntax used for the search are shown in Table 1. All retrieved database results were subsequently entered into a reference management database (Citavi 6, Swiss Academic Software, Wädenswil, Switzerland), where incompletely imported data sets such as records labeled as conference proceedings (cf. inclusion criteria) were removed. Furthermore, duplicates were manually removed from the following screening and selection steps. Search reruns to check for relevant updates were carried out on April 25, 2022, and on April 25, 2023, using the same search strategy.

**Table 1:** Details of the search process for each database.

Database	Date	Access via	Browser	Limits set	Syntax	Number retrieved
PubMed	06.20.2021 (Reruns: 04.25.22/04.25.23)	https://pubmed.ncbi.nlm.nih.gov/	Firefox	English, German, all years	("binaural"[Title/Abstract] AND "beat*"[Title/Abstract] AND ("eeg"[Title/Abstract] OR "electroencephalogra*"[Title/Abstract])) AND (english[Filter] OR german[Filter])	34 (Reruns: 6/10)
Web of Science	06.20.2021 (Reruns: 04.25.22/04.25.23)	http://webofknowledge.com/WOS	Firefox	English, German, all years	(((TI = (binaural) OR AB = (binaural) OR AK = (binaural)) AND (TI = (beat*) OR AB = (beat*) OR AK = (beat*)))) AND (((TI = (eeg) OR AB = (eeg) OR AK = (eeg)) OR (TI = (electroencephalogra*) OR AB = (electroencephalogra*) OR AK = (electroencephalogra*))))	40 (Reruns: 7/10)
Scopus	06.20.2021 (Reruns: 04.25.22/04.25.23)	https://www.scopus.com/search/ form.uri?display = basic#basic	Firefox	English, German, all years	(TITLE-ABS-KEY (binaural) AND TITLE-ABS-KEY (beat*)) AND (TITLE-ABS-KEY (eeg) OR TITLE-ABS-KEY (electroencephalogra*)) AND (LIMIT-TO (LANGUAGE, "English") OR LIMIT-TO (LANGUAGE, "German"))	91 (Reruns: 13/13)

### Literature selection

Initially, 185 records were identified in total. The titles and abstracts of the records that remained after a cleaning of the reference database were screened for relevance for the present systematic review. In a next step, the inclusion and exclusion criteria were applied, i.e. for all records identified as potentially relevant for the review, full texts were retrieved and assessed for eligibility using the screening and selection tool. This process resulted in a sample of 15 studies that met all criteria. Additionally, a citation search was conducted using all retrieved full texts. One further study was identified but was excluded after the application of the exclusion criteria. The search reruns identified 59 recently published new records and resulted to the screening of six additional records of which four were dismissed after full text retrieval on the basis of the inclusion and exclusion criteria. The remaining two studies were included in the review. Overall, these first identification and screening steps resulted in 17 studies that were included at this point.

An independent rater (ESP) carried out the search a second time and also reviewed the retrieved records using the screening and selection tool. The results of the two independent search processes were compared and differences were resolved by the two raters through discussion.

**Fig 1:**
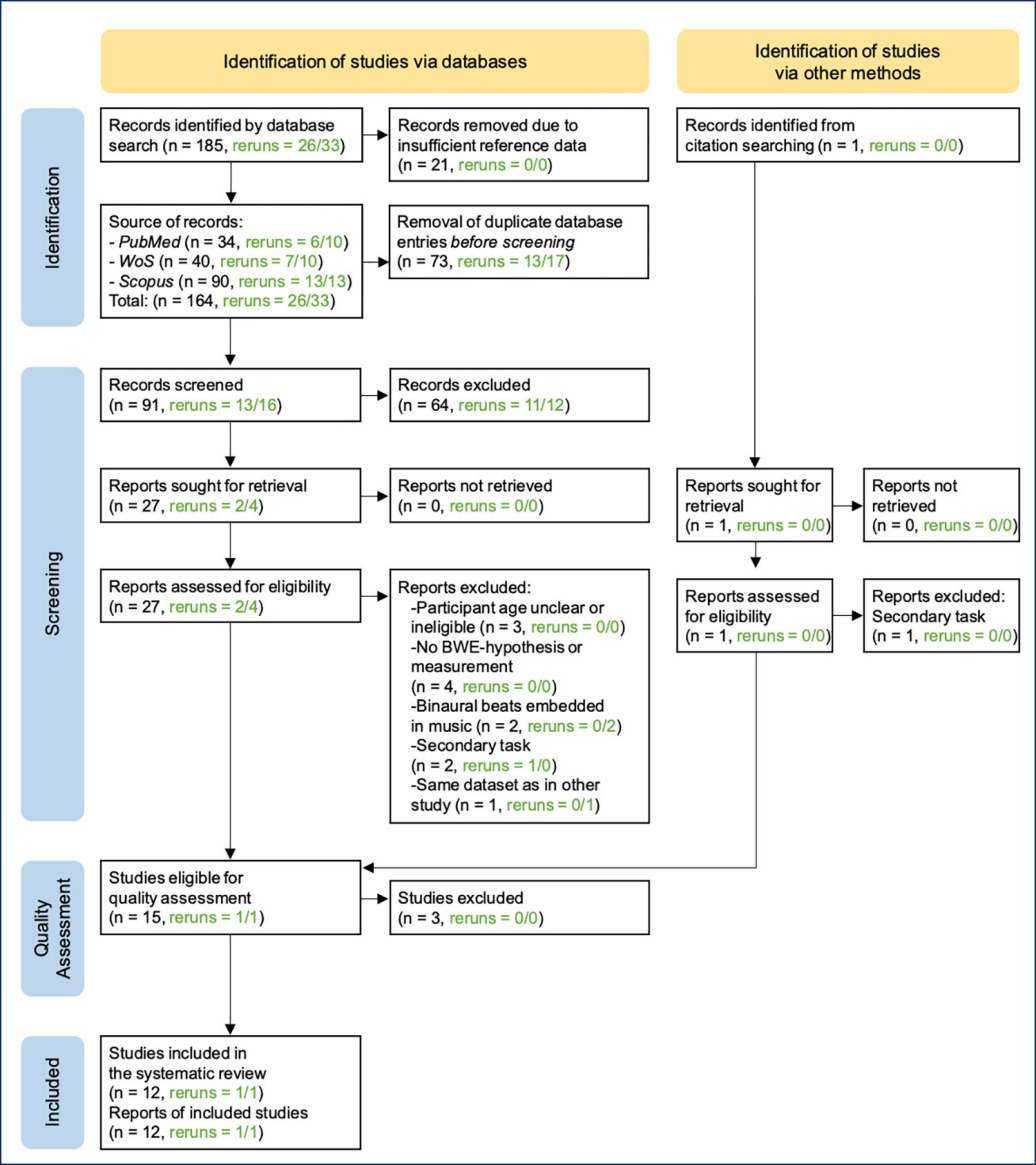
PRISMA flow diagram for the search process. (*Descriptive caption*: A flow diagram illustrates the search process, namely inputs and outcomes of the identification, screening and quality assessment phases. Described is a sequence of steps which started with 185 records identified in the initial search and additional 59 studies from search reruns, and which resulted in the 14 studies that were included in the present systematic review).

Fig 1 depicts an outline of the search process using a flow diagram in accordance with the *Preferred Reporting Items for Systematic reviews and Meta-Analyses* (PRISMA) guidelines [67]. The PRISMA checklist [68] is to be found in the supplementary documents (see S1 Table). For the present systematic review no protocol was registered.

## Results

### Quality assessment

#### General assessment of study quality

An assessment of the quality of the sample of studies was carried out. Given that the majority of the studies implemented a quasi-experimental study design, the *Checklist for Quasi-Experimental Studies* by the *Joanna Briggs Institute* [69] was used. The results of the quality assessment are shown in Table 2.

**Table 2:** Results of quality assessment of included reports.

Author(s) (Year)	Clear separation of causes and effects	Matched participants in the experimental groups	Similar treatment of the different experimental groups	Use of control group(s)	Multiple pre- and post-measurements	Complete follow-up	Outcomes measured consistently across the experimental conditions	Outcomes measured reliably	Appropriate statistical analysis
Brady & Stevens (2000)	✓	✓	✓	✓	✓	NA	✓	✖	✓
Corona-González et al. (2021)	✓	✓	✓	✖	✖	NA	✓	✓	✓
Crespo et al. (2013)	✓	✓	✓	✓	✖	NA	✓	✓	✓
da Silva Junior et al. (2018)	✓	✓	✓	✖	✖	NA	✓	✓	✓
Deraman & David (2017)	✓	✓	✓	✖	✖	NA	✓	U	U
Gao et al. (2014)	✓	✓	✓	✖	✖	NA	✓	✓	✓
Ioannou et al. (2015)	✓	✓	✓	✖	✖	NA	✓	✓	✓
Jirakittayakorn & Wongsawat (2017)	✓	U	✓	✓	✖	NA	✓	✓	✓
Kim et al. (2023)	✓	✓	✓	✖	✖	NA	✓	✓	✓
Jirakittayakorn & Wongsawat (2017b)	✓	✓	✓	✖	✖	NA	✓	✓	✓
López-Caballero & Escera (2017)	✓	✓	✓	✖	✖	NA	✓	✓	✓
Orozco Perez et al. (2020)	✓	✓	✓	✖	✖	NA	✓	✓	✓
Seifi Ala et al. (2018)	✓	✓	✓	✖	✖	NA	✓	✓	✓
Solca et al. (2016)	✓	✓	✓	✖	✖	NA	✓	✓	✓
Stevens et al. (2003)	✓	✓	✓	✓	✓	NA	✓	✖	✓
Vernon et al. (2014)	✓	✓	✓	✖	✖	NA	✓	✓	✓
Wahbeh et al. (2007)	✓	✓	✓	✖	✖	NA	✓	✓	✓

✓ = Yes (applied); ✖ = No (not applied); U = Unclear (not stated); NA = Not applicable.

Overall, while the quality of the final sample of studies was mixed, a consistent pattern of strengths and shortcomings was discernible. All studies that met the inclusion criteria had implemented study designs that allow for a differentiation of causes and effects, i.e. the binaural beat stimulation was applied as a treatment, and resulting changes in EEG parameters were examined as outcome variables. With the exception of one study that did not explicate the assignment procedure [43], participants in the treatment or control groups were comparable in terms of demographics. This applies, however, only to the six studies that assigned different groups of participants to treatment or control conditions. The majority of the studies used within-subject designs, where experimental groups are matched in principle [69]. Across the studies, treatment outcomes were predominantly measured, analyzed and assessed in a transparent and consistent manner for all experimental conditions. Only the study by Deraman and David [70] did not explicitly state specifics of EEG data collection or details of the data analysis which makes a comparison with the other studies difficult. In addition, while the studies conducted by Brady and Stevens [71] and by Stevens et al. [53] used EEG data acquisition methods adequate to their time of publication, they are not meeting current methodological standards.

The quality assessment also pointed to certain shortcomings across the sample of studies. As previously mentioned, twelve out of the 16 studies did not implement a control group design. Furthermore, only two studies [53, 71] conducted multiple pre- and post-measurements so as to investigate effects which are not immediately attributable to the intervention [69]. These systematic shortcomings related to the study designs make causal inferences difficult to ascertain.

#### EEG-specific methodological assessment

Given that the procedures related to the collection, preprocessing and analysis of EEG data vary across the studies, the sample was assessed with respect to the reported EEG measurement approaches. The two oldest studies [53, 71] were regarded as not appropriate from a methodological point of view (i.e. single EEG channel, inappropriate artifact-rejection procedures etc.) which led to their exclusion.

A further study [70] had to be excluded from the sample of 17 due to missing information on EEG acquisition and data analysis, i.e. parameters of the EEG system, filter adjustments as well as the approach to signal processing were not reported. Since this information is crucial for the evaluation of EEG results, an adequate level of methodological quality could not be determined for this study.

### Overall characteristics of the sample of studies

The relevant characteristics of the remaining 14 studies are presented in Table 3.

**Table 3:** Study characteristics of the included sample.

ID	Authors	Journal	Country	Research question
01	Corona-González et al. (2021)	Frontiers in Psychology	Mexico	Effects of stimulation using personalized theta and beta binaural beats on EEG oscillatory activity
02	Crespo et al. (2013)	Archives of Acoustics	Spain	Effects of binaural beat stimulation in the theta and beta ranges on EEG parameters and on attention
03	Da Silva Junior et al. (2019)	Cognitive Systems Research	Brazil	Effects of a binaural beat stimulation on EEG frequency measures before and after stimulus presentation
04	Gao et al. (2014)	International Journal of Psychophysiology	China	Effects of extended binaural beat presentation on EEG oscillatory activity and connectivity
05	Ioannou et al. (2015)	PloS One	England	EEG effects of short binaural beat presentation on EEG responses of musicians and non-musicians
06	Jirakittayakorn & Wongsawat (2017a)	International Journal of Psychophysiology	Thailand	Effects of binaural beats on EEG oscillatory activity, working memory and emotional states
07	Jirakittayakorn & Wongsawat (2017b)	Frontiers in Neuroscience	Thailand	Effects of a binaural beat on EEG theta activity
08	Kim et al. (2023)	Technology and Health Care	South Korea	Gender differences in effects of binaural beat stimulation on frequency following responses
09	López-Caballero & Escera (2017)	Frontiers in Human Neuroscience	Spain	Effects of binaural beats on oscillatory activity in specific EEG bands, heart rate and skin conductance
10	Orozco Perez et al. (2020)	Cognition and Behavior	Canada	Comparison of brain responses to binaural and monaural beat stimulation on EEG oscillatory activity and connectivity as well as effects on mood
11	Seifi Ala et al. (2018)	Biomedical Signal Processing and Control	Iran	Effects of binaural beats on EEG oscillatory activity and on connectivity
12	Solca et al. (2016)	Hearing Research	Switzerland	Effects of binaural beats on hemisphere synchronization at neural and behavioral levels
13	Vernon et al. (2014)	International Journal of Psychophysiology	England	Effects of alpha and beta binaural beats on EEG oscillatory activity
14	Wahbeh et al. (2007)	The Journal of Alternative and Complementary Medicine	USA	Effects of binaural beats on EEG oscillatory activity

Studies arranged in alphabetical order.

Evidently, the scientific investigation of binaural beat stimulation in line with the question of the present review has a history of about 15 years, with the first study being published in 2007 [38] and the most recent one in 2023 [72]. The journals in which the studies are published cover a topical scope ranging from hearing research and acoustics to neuroscience and different areas of applied psychology. The studies were conducted by research groups from various countries in Europe, Asia and North- and South America, which means that groups of participants from different cultural backgrounds were involved. An examination of the research questions yielded five studies that focused on effects of binaural beat stimulation on EEG parameters in terms of classical brainwave entrainment [25, 38, 43, 52, 55]. Of the remaining studies, four stated an additional focus on connectivity measures [1, 50, 59, 62]. Six studies focused on applied effects of binaural beats on measures of attention [54], working memory and emotional states [73], mood [1] and physiological correlates [51]. One study aimed at comparing effects of binaural beat stimulations for male and female participants [72] and another study for groups of musicians and non-musicians [63].

### Study designs

Details of the study designs are presented in Table 4. Sample sizes range from four (a pilot study) [38] to 47 [73] participants which covers the usual range for EEG studies.

**Table 4:** Study designs of the included sample.

ID	Participant *N* / age	Beat frequencies	Beat presentation	Session procedure	Session design	Control conditions	Carrier tones
01	20 / 19–24	Personalized theta Personalized beta (based on HR)	Continuous, 20 min	2 sessions à 3 min baseline, 20 min BBs (session 1: theta, session 2: beta)	Passive, eyes closed	Silence (baseline)	500 Hz
02	8 / 26.6 ± 7.49	4 Hz (theta), 16 Hz (beta)	Continuous, 20 min	3 min baseline; then 3 conditions/groups: 1) BBs embedded in pink noise, 2) BBs embedded in pink noise with other carrier tones, 3) pink noise only	Passive, eyes closed	Pink noise only, varying carrier tones (3 groups)	100 Hz, 200 Hz, 250 Hz, 300 Hz, 500 Hz, 650 Hz, 750 Hz, 900 Hz
03	6 / 18–35	5 Hz (theta)	Continuous, 20 min	10 sessions: 1 & 10: baseline, then 20 min BBs, then post; 2–8: 20 min BB-Training without EEG	Passive, eyes closed	Silence (baseline)	400 Hz
04	13 / 19–26	1 Hz (delta), 5 Hz (theta), 10 Hz (alpha), 20 Hz (beta)	Continuous, 5 min, embedded in pink noise	5 min baseline (pink noise only), then 4 BB-frequencies for 5 min each + break for 2 min each (pink noise only)	Passive, eyes closed	Pink noise only	550 Hz
05	16 musicians & 16 non-musicians / 25.5 ± 3.18 & 26.1 ± 3.82	1–4 Hz (delta), 5–8 Hz (theta), 9–12 Hz (alpha), 13–30 Hz (beta), 32–48 Hz (gamma)	1 cycle = run through 1–48 Hz in 1 min (division into frequency bands afterwards)	34 blocks à 2.20 min: 20 s silence, 1 min no-BB, 1 min BB; during BB-condition gradual rise from 1–48 Hz	Silent movie, instructed to ignore sounds	Carrier tone only (no-BB condition), silence	200 Hz
06	47 / 20.6 ± 1.4	40 Hz (gamma)	Continuous, 30 min	5 min baseline, 30 min BB stimulation, 5 min post	Asked to focus on stimulus	Silence (baseline)	250 Hz
07	28 / 21.9 ± 1.9	6 Hz (theta)	Continuous, 30 min	5 min baseline, 30 min stimulation (experimental: BB, control: silence), 5 min post	Asked to focus on stimulus but randomly think	Silence (2 groups)	250 Hz
08	23 / 20–29	10 Hz (alpha)	Continuous, 5 min	5 min baseline, then 5 min BB stimulation	Not stated	Silence (baseline)	250 Hz
09	14 / 23.3 ± 3.3	4.53 Hz (theta), 8.97 Hz (alpha), 17.93 Hz (beta), 34.94 Hz (gamma), 57.3 Hz (upper gamma above BB threshold)	Continuous, 3 min, embedded in pink noise	3 h total: 10 blocks (5 BB-conditions, 5 MB-conditions) à 6 min: 90 s pink noise, 180 s stimulus, 60 s pink noise; 45–75 s breaks between each block	Silent movie	Monaural, pink noise only	373 Hz
10	16 / 27.4 ± 5.5	7 Hz (theta), 40 Hz (gamma)	Continuous, 8 min	8 min baseline, then 4 x 8 min experimental (2x BB, 2x MB à 8 min), break to rate experience after each block	Passive, eyes closed	Monaural, silence (baseline)	400 Hz
11	15 / 25.5 ± 3.5	7 Hz (theta)	Continuous, 3 min, embedded in pink noise	7 blocks: 1x baseline pink noise, 3 x (3 min BB + 1 min pink noise), 15 min total	Passive, eyes closed	Pink noise only	200 Hz
12	9 / 25–34	4 Hz (delta), 10 Hz (alpha)	Continuous, 4 min	Baseline, then 4 conditions (10 Hz BB, 4 Hz BB, 10 Hz MB, 4 Hz MB) à 4 min in random sequence	Eyes closed, asked to listen to sound	Monaural, silence (baseline)	400 Hz
13	22 / 18–32	10 Hz (alpha), 20 Hz (beta)	Continuous, 1 min	Baseline: 2 min eyes open + 2 min eyes closed, then 10 segments à (1 min BB + 1 min pure carrier tone), post: 2 min eyes open + 2 min eyes closed	Silent nature DVD	Alpha vs. beta BB (2 groups), carrier tone only	400 Hz
14	4 / 43–57	7 Hz (theta)	Continuous, 30 min, embedded in pink noise	2 sessions à 10 min baseline, 30 min stimulation/control, 15 min post	Passive, eyes closed	Pink noise only	133 Hz

Age in years; BB = binaural beat; MB = monaural beat; min = minutes; s = seconds; HR = heart rate.

Overall, the participants represent a young adult population with mean ages in the twenties and an upper age limit of around 35 years, with the exception of one study that investigated an older sample [38]. Four studies [50–52, 55] involved samples consisting of university students exclusively.

Across all studies, binaural beats in all human EEG frequency bands were investigated. Beats in the range of the theta band were implemented in ten studies, thus, being the EEG frequency used most often. Beats in the delta band were investigated in three studies. One study [51] implemented a frequency of 57.3 Hz (upper gamma band), which means that in this study the assumed threshold for binaural beat perception [6] was actually exceeded. Continuous presentation of the binaural beats was used in 13 studies, ranging from one to 30 minutes of continuous stimulation. The remaining study [63] implemented binaural beats with a periodic cycling through a certain frequency range within a minute of stimulation. Four studies embedded the binaural beats in pink noise, while ten studies presented pure binaural beats.

The most obvious differences between the 14 studies were related to the reported study designs and procedures. All studies implemented a form of baseline EEG measurement before stimulus presentation. Post EEG measurement after stimulus presentation was reported in eight studies. The durations of pre- and post-measurements ranged from 20 seconds to ten minutes, during which participants were presented either with silence or with pink noise. The majority of the studies collected EEG data within a single session with a maximum duration of three hours. Three studies implemented multiple, i.e., between two and ten EEG sessions [25, 38, 55]. Among them, one study collected EEG data only during the first and last of ten stimulation sessions [25]. An additional difference in designs was the use of comparison groups which was done in four studies. The remaining ten studies relied on within-person comparisons. The procedures differed further in terms of breaks between stimulation sessions and, most importantly, the arrangement of stimulation conditions, i.e., application of different binaural beat frequencies or contrasting binaural beats with, for example, monaural beats. Participants were asked to listen passively to the stimuli with their eyes closed in the majority of the studies. Exceptions were studies that had participants watch a silent movie [51, 52, 63], focus on or listen to the sound [43, 62, 73], or try to ignore the presented sounds actively [63]. One study did not give information on the instructions given to the participants [72]. The carrier tones used in the sample ranged from 100 Hz to 900 Hz and were, thus, all below the threshold of 1 kHz [4]. Only four studies used the 400 Hz carrier frequency [1, 25, 52, 62] which was suggested by Goodin et al. [5] to yield optimal effects in binaural beat stimulation.

### Data analysis and study results

An overview of the reported results of the 14 studies is presented in Table 5.

**Table 5:** Study results of the included sample.

ID	EEG apparatus	Parameters operationalized as entrainment	Statistical analyses	Main findings
01	24 channels (mBrainTrain)	Absolute power, relative power (for each frequency band)	Paired t-test (baseline vs. theta/beta stimulation), two-way ANOVA for each frequency band: relative power value, channel, band	Absolute power differences in response to theta and beta BB stimulation compared to the baseline condition; no significant differences between the theta and the beta sessions in the respective EEG frequency bands
02	29 scalp points (Brainvision Braimnamp)	Ratio of power between stimulation and baseline for each frequency band	Non-parametric Kruskal-Wallis analysis of variance	No significant differences between three experimental groups for any electrode site, frequency band, or period of stimulation
03	19 active electrodes (Nexus-32)	Pre-post statistical analysis of amplitudes for different EEG frequency bands, source localization	Wilcoxon-Test	No significant changes in the stimulated EEG theta frequency band
04	18 electrodes (Neuroscan Company)	Relative power (for each frequency band)	Paired t-test (resting state vs. stimulation)	No increase in relative power in the respective EEG frequency band in response to BBs in the delta, theta, alpha and beta ranges
05	64 active electrodes (Biosemi)	Normalized spectral power, ASSR (estimated by averaging the EEG spectral power over stimulated frequency band when stimulated with the respective frequency)	Factorial ANOVA (between: musicians vs. non-musicians, within: alpha vs. gamma)	Increased alpha band power during alpha-BB stimulation; alpha power increase during delta BB stimulation (cross frequency response)
06	Elastic cap with mesh electrodes (BrainMaster)	Absolute power of gamma and beta oscillations	Paired t-tests (baseline vs. each interval)	Increased gamma power in temporal, frontal, and central regions in response to gamma BB stimulation
07	Elastic cap with mesh electrodes (BrainMaster)	Absolute power of theta activity	Paired t-tests (baseline vs. each interval) within groups, independent t-tests between groups	Increased theta power at almost all electrode sites in the experimental group in response to theta BB stimulation, no significant frequency effects in the control condition
08	19 channels (Enobio20)	Mean absolute power of the alpha frequency band	Mixed-design ANOVA with conditions stimulation (resting state/BB), gender (male/female), brain area (5 areas)	Increase in absolute alpha power during the stimulation phase compared to the resting phase with exception of the temporal area; no differences in gender
09	36 scalp electrodes (Quickcap)	Normalized EEG spectral power, scalp distribution of EEG spectral power	Repeated measures ANOVA for each frequency: session (pre/beat/post), treatment (BB/MB), electrode (15 levels)	No significant effects in EEG spectral power within the theta, alpha, beta or gamma frequency ranges for BB stimulation in the respective frequency bands; no effects in response to monaural beat (MB) stimulation
10	46 electrodes (ActiveTwo)	FFR, ASSR (normalized power¸ differing in filtering process, ICA decomposition and re-referencing)	Repeated measures ANOVA: beat type (BB vs. MB), frequency (theta, gamma)	Similar FFRs at the respective carrier frequencies in response to theta and gamma BBs and MBs; ASSRs in response to theta BBs and MBs with responses to MBs peaking higher than to BBs; ASSRs in response to gamma BBs and MBs with responses to BBs peaking higher than to MBs
11	19 active electrodes (g.Hlamp)	Absolute power, relative power	Repeated measures ANOVAs, Sidak adjustment	Relative theta power changes for the parietal and temporal electrode sites in response to theta stimulation; no significant changes in absolute power for different frequency bands for any scalp region after Sidak correction
12	128 channels (Biosemi)	Difference in power between resting state, MB and BB in the Heschl Gyrus	Paired t-tests (resting state vs. BB vs. MB)	No significant increase in band power in response to alpha and theta BBs compared to a resting condition or the MBs in the respective frequencies
13	2 sensors (Nexus-10 DC coupled portable)	Mean and peak amplitudes for alpha and beta frequency bands	Repeated measures ANOVA for each frequency band: time (1 min-10 min), hemisphere (left/right), signal (BB/BB off)	Exposure to interleaved epochs of alpha or beta BB stimulation resulted in no significant frequency effects
14	32 channels (Active 2, Biosemi)	Total theta power, peak frequency (largest magnitude) between 2–20 Hz	Independent sample t-tests	Larger changes from baseline level in theta band power for the control compared to the BB condition; no significant changes in theta power in response to BB stimulation compared to the baseline condition

BB = binaural beat; ASSR = Auditory Steady State Response; FFR = Frequency Following Response; min = minutes.

In terms of the EEG parameters which were used to operationalize brainwave entrainment, a number of studies referred to absolute power [38, 43, 55, 59, 72, 73], while other studies reported relative [50, 55, 59] or normalized power measures [1, 51, 63]. Three studies implemented ASSR and FFR measures [1, 63, 72], and one study used EEG source localization techniques [25].

For the majority of the studies, the statistical analyses focused on comparisons over time, i.e., either repeated measures ANOVAs or paired t-tests were used to compare EEG parameters for baseline and experimental conditions and, where applicable, post-measurement. Two studies comparing either different experimental groups [63] or different sessions [38] applied between-group comparisons. The remaining studies used non-parametric tests for comparisons [25, 54].

With respect to the study outcomes, only six out of the 14 studies of the final sample reported results in line with the entrainment hypothesis. Enhancement of EEG power through binaural beat stimulation when compared to control conditions or baseline recordings was found for the theta [1, 43, 59], the alpha [63, 72] and the gamma bands [1, 73]. The required stimulation durations were either six or ten minutes for theta [43, 59], five minutes for alpha [72], and 15 minutes for gamma entrainment [73]. Entrainment effects were discernible at parietal and temporal electrode sites for the theta band [59], at frontal, central, parietal and occipital areas for the alpha band [72], and at temporal, central and frontal scalp regions for the gamma band [73]. Comparisons with monaural beats revealed a higher ASSR for theta monaural beats compared to theta binaural beats, and a reversed pattern for gamma monaural and binaural beats, while no difference in FFR was found for the comparison of binaural and monaural beats [1]. One study additionally investigated cross-frequency responses and reported enhanced alpha power in response to a delta binaural beat stimulation [63].

Of the 14 studies, nine reported results that are at least partially inconsistent with the assumption of brainwave entrainment. Among these, one study reported mixed results [59] and is therefore mentioned both in support and against the BWE hypothesis, i.e., the authors found changes in relative theta power in response to theta binaural beats, but no corresponding changes in absolute power. The remaining eight studies reported no differences in EEG oscillatory activity in response to binaural beat stimulation compared to baseline or control conditions for the theta [25, 38, 50, 51, 54, 59, 62], the alpha [50–52, 62], the beta [50–52, 54] and the gamma bands [51]. While Corona-Gonzàlez et al. [55] demonstrated a generally increased absolute EEG power during binaural beat stimulation compared to the resting state condition, no frequency-specific effects were found for stimulations in the theta or the beta frequency, respectively. These results were, thus, contradictory to the assumption of differential entrainment effects in EEG bands in response to the respective binaural beat stimulation. Furthermore, one study failed to find entrainment effects even for the monaural beat conditions [51], and another study reported increased theta power for a pink noise-only compared to the binaural beat condition [38]. For these nine studies, the reported stimulation durations ranged from one to 30 minutes and comprised analyses of EEG parameters for different scalp regions. No systematic similarities in the designs of the studies were obvious, apart from the fact that all studies that embedded the binaural beats in pink noise are among those studies not finding entrainment.

Due to the heterogeneity in study designs as well as EEG measurement and analyses, and in the statistical procedures, the outcomes of the sample of 14 studies were not deemed qualified for a meta-analysis. A quantitative evaluation of the results was, thus, not further pursued.

## Discussion

The present systematic review aims at an evaluation of available empirical studies investigating brainwave entrainment effects in response to binaural beat stimulation. To examine this question, a systematic search for literature was performed, and a selection process according to specified inclusion and exclusion criteria was conducted. The quality of the available studies was assessed subsequently. In a last step, information concerning study characteristics, study designs and study results was extracted and evaluated. Our findings demonstrate not only contradictory study outcomes, but, most notably, a considerable heterogeneity in study approaches in this field of research. As evident from the present review, the lack of an established general methodological framework renders the outcomes of the available studies of limited comparability.

Specific effects of binaural beat stimulation on EEG parameters have been a research topic for almost the last two decades and have been investigated by a number of independent research groups. The included studies involved small samples of young adults, and applied binaural beat stimulation in the main human EEG frequency bands. The studies varied considerably with respect to their designs, procedures, measurement approaches, EEG parameters and analyses.

The synthesis revealed contradictory results, with five studies reporting results in line with the brainwave entrainment hypothesis [15]. Eight studies reported no effects in terms of entrainment, and one study reported mixed results. Entrainment effects in response to binaural beat stimulation compared to control conditions was found for the theta, alpha, and gamma bands, while none of the studies using stimulation in the beta frequency range found entrainment effects.

Overall, there is no clear trend discernible either in favor of the brainwave entrainment hypothesis or against it. Two aspects should be mentioned at this point, however. At least with respect to the studies included in the present systematic review, the majority reported negative results. Technically, this casts serious doubts on whether the brainwave entrainment hypothesis is scientifically tenable. However, the second aspect of relevance in this context refers to the study conducted by Seifi Ala et al. [59] which reported mixed results only. A reanalysis of the same dataset by Shamsi et al. [74] using state-of-the-art approaches to EEG frequency analysis (*Higuchi Fractal Dimension*) yielded results in favor of the assumption of brainwave entrainment in response to binaural beat stimulation. Given the striking methodological heterogeneity of the studies included in our review, this latter finding suggests that the inconsistency in research outcomes might be at least partially attributable to specifics in the approaches to EEG measurement and analysis.

Consequently, while the present systematic review cannot provide an unequivocal answer to the research question of whether stimulation with binaural beats in the frequency range of the human EEG elicits brainwave entrainment effects, it certainly emphasizes the need for further systematic and more standardized approaches to basic research in this field of study.

There are several aspects for methodological improvement which can be inferred on the basis of the present systematic review. First, the quality assessment of the set of studies yielded mixed results. Despite common strengths in the implementation of the binaural beat stimulation, the prevailing lack of comparison groups and of multiple pre- and post-measurements is conspicuous, and cannot be explained in view of the current state of the art in EEG measurement [cf. e.g., 75]. Future studies investigating the effects of binaural beat stimulation using EEG should, therefore, address these basic design considerations thoroughly.

Second, the limited age-range of the groups of participants casts the generalizability of the results across age groups into doubt. Even though there are no theoretical grounds to assume that binaural beat perception changes in the course of development [9], a close examination of the available literature points to an unsatisfactory dearth of studies on both younger and older groups of participants.

Third, while synthesizing the sample of studies highlighted the overall diversity in research designs, certain patterns of consistency in the implementations and related studies outcomes are discernible nonetheless. For example, the fact that all studies embedding binaural beats in pink noise reported no effects in line with the brainwave entrainment hypothesis, could be a motivation to investigate the impact of the embedding of binaural beats in pink noise in contrast to other noise conditions—all the more so in view of past research that suggests binaural beat perception to be enhanced through noise embedding [9].

Fourth, EEG parameters which were predominantly used to assess entrainment fall within the broader category of EEG-power measures. Yet, given that we do not have a generally accepted definition of what brainwave entrainment in response to binaural beat stimulation actually is, the question of operationalization is not settled in principle. This points to a need for a comprehensive theoretical and methodological debate about the definition of brainwave entrainment which should result in an alignment with respect to EEG measures to assess entrainment effects.

Finally, and most importantly, the present synthesis of findings in basic research has implications for studies on binaural beats in the more applied fields in cognitive neuropsychology. The apparent lack of a sound empirical basis for the assumed electrophysiological underpinnings of higher-level changes in psychological outcomes (e.g., changes in mood [39] or cognitive processing [34] through binaural beat stimulation) challenges the results of a number of studies that merely assumed brainwave entrainment effects without actually measuring them [36, 37, 40, 42, 44–46]. As pointed out by Lersch et al. [56], future studies on psychological or physiological effects of binaural beat stimulation have to incorporate neurocognitive measurement to empirically ascertain brainwave entrainment in a first step, before investigating changes in mental or physiological states that are assumed to be causally dependent on changes in the respective EEG parameters.

### Limitations

This systematic review is, to the authors’ knowledge, the first to systematically synthesize and evaluate existing research on the question of whether binaural beat stimulation can be assumed to elicit brainwave entrainment effects. Even though compliant with the PRISMA guidelines for systematic reviews [67], a number of limitations should be pointed out:

First, the fact that only published studies that met the set of inclusion criteria were incorporated in this review must be taken into consideration. Unpublished research, including grey literature, was not surveyed, but excluded from the outset. This deliberate decision was the result of the outcomes of scoping searches and aimed at providing a body of empirical evidence satisfying rigorous scientific quality criteria. Nevertheless, it should be kept in mind that the range of output from this field of research might not have been captured in its entirety.

Second, it should be noted that while most parts of the review process including the search and selection of the literature and the EEG-specific methodological assessment were executed in parallel by independent researchers, the data extraction was carried out by only one of the authors (RMI) due to group-internal decisions. However, extracting and synthesizing data by different researchers independently can be assumed to further reduce bias, thus making the results more robust in principle.

Third, the study outcomes extracted for this review did not allow for further quantitative analyses. This is due to the considerable methodological heterogeneity of the 14 studies in the sample, which did not provide sufficient similarity for an analysis of quantitative data. Consequently, this review can only provide a synthesis of results on a descriptive level. Deeper insights in terms of effect sizes cannot be inferred.

Last, it should be noted that this systematic review was not preregistered. Doing so is generally desired in order to enhance the power of study results. However, the availability of a comprehensive process documentation and of the full dataset allows for complete transparency regarding the present systematic review and, thus, its outcomes.

### Directions for future research

As a result of our systematic review, a number of starting points for future research become evident. First and foremost, the research question of whether brainwave entrainment in response to binaural beat stimulation can be assumed, remains open. Further research conducted in a consistent manner is necessary in order to provide a well-founded answer to that question. There is a need for a certain level of standardization with respect to the methodological basis in order to ensure comparability of study results. This implies a commonly accepted definition of the concept of brainwave entrainment in the first place. Only when a consensus is reached with respect to the methodological basis should EEG studies be implemented following predefined protocols in order to tackle the open issues of brainwave entrainment in response to binaural beat stimulation in general and, subsequently, of specific differences in binaural beat perception for groups of participants and under various experimental conditions.

## Conclusion

The research question of this systematic review is whether there is sufficient empirical evidence for the assumption of brainwave entrainment effects due to binaural beat stimulation. To answer this question, the available literature reporting basic research studies on the effects of binaural beats on EEG parameters was screened and selected according to predefined inclusion and exclusion criteria. Fourteen studies met the inclusion criteria and passed the quality assessment. The review yielded inconsistent findings with five studies reporting results in line with the brainwave entrainment hypothesis, eight studies reporting contradictory results, and one mixed results. The synthesis of the 14 studies revealed substantial methodological heterogeneity, limiting the comparability of the study results. Consequently, the research question cannot not be settled at this point. Still, this review provides a relevant contribution to the field of research by pointing out the need for a wider scientific discourse about the definition and an operationalization of brainwave entrainment in a first step, in order to allow for a degree of methodological homogenization and standardization in a second step. Furthermore, the present systematic review highlights the fact that existing studies reporting changes in psychological and physiological functioning due to putative effects of brainwave entrainment in response to binaural beat stimulation should be considered with caution.

## Supporting information

S1 Checklist PRISMA checklist. (DOC)

S1 Table Search record. For transparency reasons, an outline of the screening and selection process is provided for every located record.
(DOCX)
