# Supplementary material for: Binaural beats to entrain the brain? A systematic review of the effects of binaural beat stimulation on brain oscillatory activity, and the implications for psychological research and intervention
Source: PLoS One. 2023 May 19;18(5):e0286023. doi: 10.1371/journal.pone.0286023 (PMC10198548; doi:10.1371/journal.pone.0286023)
Supplement: S1 Table — For transparency reasons, an outline of the screening and selection process is provided for every located record. (DOCX) [file pone.0286023.s002.docx]

**Search Record Table**

| **Reference** | **Database** | **Research Type** | **Included at screening?** | **Obtained paper through** | **Included at selection?** | **Reason for exclusion** |
| --- | --- | --- | --- | --- | --- | --- |
| Abeln V, Kleinert J, Strüder HK, Schneider S. Brainwave entrainment for better sleep and post-sleep state of young elite soccer players - A pilot study. European journal of sport science. 2014;14(5):393–402. doi: 10.1080/17461391.2013.819384. | Scopus | Journal Article | No |  |  | No EEG measures to validate BWE |
| Ali AJM, Al-Obaidi FEM. Gamma binaural beats and its quality assessment. Research journal of pharmacy and technology. 2018;11(11):4842–5. doi: 10.5958/0974-360x.2018.00880.6. | Scopus | Journal Article | No |  |  | Wrong study goal |
| Amarasena J, Indimagedara N, Wanigasuriya S, Bandare B, Pulasinghe K, Tharmaseelan J. IPillow: Sleep quality improvement system. 2019. p. 427–33. | Scopus | Proceedings Paper | No |  |  | Wrong research type (conference) |
| Anninos PA, Anogianakis G, Lehnertz K, Pantev C, Hoke M. Biomagnetic measurements using squids. The International journal of neuroscience. 1987;37(3-4):149–68. doi: 10.3109/00207458708987144. | Scopus | Journal Article | No |  |  | MEG not EEG |
| Bang YR, Choi HY, Yoon I-Y. Minimal effects of binaural auditory beats for subclinical insomnia: A randomized double-blind controlled study. Journal of clinical psychopharmacology. 2019;39(5):499–503. doi: 10.1097/jcp.0000000000001097. | Scopus | Journal Article | No |  |  | BBs embedded in music |
| Beauchene C, Abaid N, Moran R, Diana RA, Leonessa A. The effect of binaural beats on visuospatial working memory and cortical connectivity. PloS one. 2016;11(11). doi: 10.1371/journal.pone.0166630. | Scopus | Journal Article | No |  |  | Secondary task |
| Beauchene C, Abaid N, Moran R, Diana RA, Leonessa A. The effect of binaural beats on verbal working memory and cortical connectivity. Journal of neural engineering. 2017;14(2). doi: 10.1088/1741-2552/aa5d67. | Scopus | Journal Article | No |  |  | Secondary task |
| Beauchene C, Leonessa A, Roy S, Simon J, Abaid N, editors. Closed-loop control of the frequency response of the virtual brain model. ASME 2017 Dynamic Systems and Control Conference, DSCC 2017; 2017. | Scopus | Proceedings Paper | No |  |  | Wrong research type (conference) |
| Becher A-K, Hoehne M, Axmacher N, Chaieb L, Elger CE, Fell J. Intracranial electroencephalography power and phase synchronization changes during monaural and binaural beat stimulation. The European journal of neuroscience. 2015;41(2):254–63. doi: 10.1111/ejn.12760. | WoS | Journal Article | No |  |  | Wrong sample (epilepsy patients) |
| Bhattacharya J, Pereda E, Ioannou C. Functional associations at global brain level during perception of an auditory illusion by applying maximal information coefficient. Physica A: Statistical mechanics and its applications. 2018;491:708–15. doi: 10.1016/j.physa.2017.09.037. | Scopus | Journal Article | Yes | sciencedirect | No | No bwe-hypothesis or measurement |
| Bischoff P, Plümer L, Scholz J, Drögemeier K, Knobelsdorff Gv, Am Schulte Esch J. Effect of remifentanil on clinical and electroencephalographic parameters of depth of anesthesia in balanced anesthesia with propofol, enflurane or isoflurane. Anaesthesiologie und Reanimation. 1998;23(5):116–23. PubMed Central PMCID: PMC9854329. | Pubmed | Journal Article | No |  |  | Wrong study goal |
| Brady B, Stevens L. Binaural-beat induced theta EEG activity and hypnotic susceptibility. The American journal of clinical hypnosis. 2000;43(1):53–69. doi: 10.1080/00029157.2000.10404255. | WoS | Journal Article | Yes | University library system | Yes |  |
| Calomeni MR, Da Furtado Silva V, Velasques BB, Feijó OG, Bittencourt JM, Ribeiro de Souza E Silva AP. Modulatory effect of association of brain stimulation by light and binaural beats in specific brain waves. Clinical practice and epidemiology in mental health : CP & EMH. 2017;13:134–44. doi: 10.2174/1745017901713010134. PubMed Central PMCID: PMC29238390. | Pubmed | Journal Article | No |  |  | Wrong sample (children, diseases) |
| Carrick FR, Pagnacco G, Hankir A, Abdulrahman M, Zaman R, Kalambaheti ER, et al. The treatment of autism spectrum disorder with auditory neurofeedback: A randomized placebo controlled trial using the Mente Autism device. Frontiers in neurology. 2018;9(JUL). doi: 10.3389/fneur.2018.00537. | Scopus | Journal Article | No |  |  | Wrong sample (autism) |
| Chaieb L, Wilpert EC, Hoppe C, Axmacher N, Fell J. The impact of monaural beat stimulation on anxiety and cognition. Frontiers in human neuroscience. 2017;11. doi: 10.3389/fnhum.2017.00251. | Scopus | Journal Article | No |  |  | MBs not BBs, no EEG |
| Chaieb L, Wilpert EC, Reber TP, Fell J. Auditory beat stimulation and its effects on cognition and mood states. Frontiers in psychiatry. 2015;6(MAY). doi: 10.3389/fpsyt.2015.00070. | Scopus | Journal Article | No |  |  | Wrong research type (review) |
| Crespo A, Recuero M, Galvez G, Begoña A. Effect of binaural stimulation on attention and EEG. Archives of acoustics. 2013;38(4):517–28. doi: 10.2478/aoa-2013-0061. | Scopus | Journal Article | Yes | journals.pan.pl (Polska Akademia Nauk) | Yes |  |
| Cvetkovic D, Djuwari D, Cosic I. The induced rhythmic oscillations of neural activity in the human brain. 2004. p. 306–11. | Scopus | Proceedings Paper | No |  |  | Wrong research type (conference) |
| Da Silva Junior M, Freitas RCd, dos Santos WP, Azevedo da Silva WW, Araujo Rodrigues MC, Quintas Conde EF. Exploratory study of the effect of binaural beat stimulation on the EEG activity pattern in resting state using artificial neural networks. Cognitive systems research. 2019;54:1–20. doi: 10.1016/j.cogsys.2018.11.002. | Scopus | Journal Article | Yes | researchgate | Yes |  |
| Deraman SN, David NV. Gamma-domain brainwave stimulation using isochronic tones. Journal of science and technology in the tropics. 2017;13(2):75–84. | Scopus | Journal Article | Yes | University library system | Yes |  |
| Derner M, Chaieb L, Surges R, Staresina BP, Fell J. Modulation of item and source memory by auditory beat stimulation: A pilot study with intracranial EEG. Frontiers in human neuroscience. 2018;12. doi: 10.3389/fnhum.2018.00500. | Scopus | Journal Article | No |  |  | Wrong sample (epilepsy patients) |
| Friedrich W, Du S, Balt K. Studying frequency processing of the brain to enhance long-term memory and develop a human brain protocol. Technology and health care. 2015;23:S465-S71. doi: 10.3233/thc-150983. | Scopus | Proceedings Paper | No |  |  | Wrong research type (conference) |
| Gabbualoy S, Punkabut S, Phasukkit P, Tungjitkusolmun S. Laboratory design for reduce brainwaves frequency by using monaural beats. BMEiCON 2019 - 12th biomedical engineering International conference; 2019. | Scopus | Proceedings Paper | No |  |  | Wrong research type (conference) |
| Gálvez G, Recuero M, Canuet L, Del-Pozo F. Short-term effects of binaural beats on EEG power, functional connectivity, cognition, gait and anxiety in parkinson's disease. International journal of neural systems. 2018;28(5). doi: 10.1142/s0129065717500551. | Scopus | Journal Article | No |  |  | Wrong sample (Parkinsons patients) |
| Gantt MA, Dadds S, Burns DS, Glaser D, Moore AD. The effect of binaural beat technology on the cardiovascular stress response in military service members with postdeployment stress. Journal of nursing scholarship. 2017;49(4):411–20. doi: 10.1111/jnu.12304. | Scopus | Journal Article | No |  |  | No EEG |
| Gao X, Cao H, Ming D, Qi H, Wang X, Wang X, et al. Analysis of EEG activity in response to binaural beats with different frequencies. International journal of psychophysiology : official journal of the International Organization of Psychophysiology. 2014;94(3):399–406. doi: 10.1016/j.ijpsycho.2014.10.010. | Scopus | Journal Article | Yes | Elsevier | Yes |  |
| Gkolias V, Amaniti A, Triantafyllou A, Papakonstantinou P, Kartsidis P, Paraskevopoulos E, et al. Reduced pain and analgesic use after acoustic binaural beats therapy in chronic pain - A double-blind randomized control cross-over trial. European journal of pain (United Kingdom). 2020;24(9):1716–29. doi: 10.1002/ejp.1615. | WoS | Journal Article | No |  |  | Wrong sample (chronic pain) |
| Goodin P, Ciorciari J, Baker K, Carey A-M, Harper M, Kaufman J. A high-density EEG investigation into steady state binaural beat stimulation. PloS one. 2012;7(4):e34789. doi: 10.1371/journal.pone.0034789. | Scopus | Journal Article | No |  |  | Secondary task |
| Grose JH, Mamo SK. Electrophysiological measurement of binaural beats: Effects of primary tone frequency and observer age. Ear and hearing. 2012;33(2):187–94. doi: 10.1097/AUD.0b013e318230bbbd. | Scopus | Journal Article | Yes | ncbi.nlm.nih.gov | No | No bwe-hypothesis |
| Gupta A, Ramdinmawii E, Mittal VK. Significance of alpha brainwaves in meditation examined from the study of binaural beats. 2016. p. 484–9. | Scopus | Proceedings Paper | No |  |  | Wrong research type (conference) |
| Guruprasath G, Gnanavel S. Effect of continuous and short burst binaural beats on EEG signals. ICIIECS 2015 - 2015 IEEE International conference on innovations in information, embedded and communication systems; 2015. | Scopus | Proceedings Paper | No |  |  | Wrong research type (conference) |
| Ioannou CI, Pereda E, Lindsen JP, Bhattacharya J. Electrical brain responses to an auditory illusion and the impact of musical expertise. PloS one. 2015;10(6). doi: 10.1371/journal.pone.0129486. | Scopus | Journal Article | Yes | PlosOne | Yes |  |
| Irsyad M, Widiyanti P, Rahmatillah A. Audio-visual stimulation for improving sleep quality. AIP conference proceedings; 2020. | Scopus | Proceedings Paper | No |  |  | Wrong research type (conference) |
| Jirakittayakorn N, Wongsawat Y. The brain responses to different frequencies of binaural beat sounds on QEEG at cortical level. Annual International conference of the IEEE engineering in medicine and biology society IEEE. 2015;2015:4687–91. doi: 10.1109/embc.2015.7319440. | Scopus | Proceedings Paper | No |  |  | Wrong research type (conference) |
| Jirakittayakorn N, Wongsawat Y. Brain responses to a 6-Hz binaural beat: Effects on general theta rhythm and frontal midline theta activity. Frontiers in neuroscience. 2017;11(JUN). doi: 10.3389/fnins.2017.00365. | Scopus | Journal Article | Yes | Frontiers in Neuroscience | Yes |  |
| Jirakittayakorn N, Wongsawat Y. Brain responses to 40-Hz binaural beat and effects on emotion and memory. International journal of psychophysiology : official journal of the International Organization of Psychophysiology. 2017;120:96–107. doi: 10.1016/j.ijpsycho.2017.07.010. | WoS | Journal Article | Yes | Elsevier | Yes |  |
| Jolly JM, Komathi K, Tamilselvi R, Iswariya R, Mohanram S. Stress removal and night drive accident prevention using embedded system. International journal of control theory and applications. 2016;9(9):4137–43. | Scopus | Journal Article | No |  |  | Wrong study goal |
| Kasprzak C. Influence of binaural beats on EEG signal. Acta Physica Polonica a. 2011;119(6 A):986–90. doi: 10.12693/APhysPolA.119.986. | Scopus | Journal Article | Yes | assets.thegrommet.com | No | Age unclear, no clear bwe-hypothesis |
| Kyon D-H, Lee J-H, Bae M-J. Acoustic characteristics of sound sources for EEG turning. Information (Japan). 2014;17(12B):6505–10. | Scopus | Journal Article | No |  |  | Wrong study goal |
| Lane JD, Kasian SJ, Owens JE, Marsh GR. Binaural auditory beats affect vigilance performance and mood. Physiology & behavior. 1998;63(2):249–52. doi: 10.1016/s0031-9384(97)00436-8. | Scopus | Journal Article | No |  |  | Secondary task |
| Lavallee CF, Koren SA, Persinger MA. A quantitative electroencephalographic study of meditation and binaural beat entrainment. Journal of alternative and complementary medicine (New York, NY). 2011;17(4):351–5. doi: 10.1089/acm.2009.0691. | Scopus | Journal Article | No |  |  | Secondary task |
| Le Scouarnec RP, Poirier RM, Owens JE, Gauthier J, Taylor AG, Foresman PA. Use of binaural beat tapes for treatment of anxiety: a pilot study of tape preference and outcomes. Alternative therapies in health and medicine. 2001;7(1):58–63. | Scopus | Journal Article | No |  |  | No EEG |
| Lee M, Song C-B, Shin G-H, Lee S-W. Possible effect of binaural beat combined with autonomous sensory meridian response for inducing sleep. Frontiers in human neuroscience. 2019;13:425. doi: 10.3389/fnhum.2019.00425. | Scopus | Journal Article | No |  |  | No EEG |
| Leeds J. An introduction to the use of therapeutic music and sound in health care: Rhythm, resonance and entrainment - The body's call for unity. American journal of acupuncture. 1996;24(4):297–308. | Scopus | Journal Article | No |  |  | Wrong research type (review) |
| Lim J-H, Kim H, Jeon C, Cho S. The effects on mental fatigue and the cognitive function of mechanical massage and binaural beats (brain massage) provided by massage chairs. Complementary therapies in clinical practice. 2018;32:32–8. doi: 10.1016/j.ctcp.2018.04.008. | Scopus | Journal Article | No |  |  | Wrong study goal |
| López-Caballero F, Escera C. Binaural beat: A failure to enhance EEG power and emotional arousal. Frontiers in human neuroscience. 2017;11. doi: 10.3389/fnhum.2017.00557. | Scopus | Journal Article | Yes | Frontiers in Human Neuroscience | Yes |  |
| Lopez-Caballero F, Escera C. BINAURAL BEATS AT 34 HZ ENHANCE EEG BRAINWAVES AT THE SAME FREQUENCY RANGE. Psychophysiology. 2017;54:S165-S. | WoS | Conference Poster | No |  |  | Wrong research type (conference) |
| Meng Q, Choa F-S, Hong E, Wang Z, Islam M. Control channels in the brain and their influence on brain executive functions. Proceedings of SPIE - The International society for optical engineering; 2014. | Scopus | Proceedings Paper | No |  |  | Wrong research type (conference) |
| Mihajloski T, Bohorquez J, Ozdamar O. Scalp topography of auditory evoked responses elicited by binaural beat illusions. 2013. p. 55–6. | Scopus | Proceedings Paper | No |  |  | Wrong research type (conference) |
| Miller C, Ying L, Yuanyuan L, Jinhui Z, Bingbing W, Ying Z. Group wisdom and omnaural perception: Discovering frequency differences by averaging the retrospective time estimates of listeners. International journal of bio-science and bio-technology. 2014;6(1):145–54. doi: 10.14257/ijbsbt.2014.6.1.16. | Scopus | Journal Article | No |  |  | Wrong study goal |
| Moridis CN, Klados MA, Kokkinakis IA, Terzis V, Economides AA, Karlovasitou A, et al. The impact of audio-visual stimulation on alpha brain oscillations: An EEG study. Proceedings of the IEEE/EMBS region 8 International conference on information technology applications in biomedicine, ITAB; 2010. | Scopus | Proceedings Paper | No |  |  | Wrong research type (conference) |
| Naraballobh J, Thanapatay D, Chinrungrueng J, Nishihara A. EEG-based analysis of auditory stimulus in a brain-computer interface. 2015 6th International conference on information and communication technology for embedded systems, IC-ICTES 2015; 2015. | Scopus | Proceedings Paper | No |  |  | Wrong research type (conference) |
| Nawaz R, Nisar H, Voon YV. The effect of music on human brain; Frequency domain and time series analysis using electroencephalogram. Ieee Access. 2018;6:45191–205. doi: 10.1109/access.2018.2855194. | Scopus | Journal Article | Yes | ieeexplore.ieee.org | No | BBs embedded in music |
| Nawaz R, Nisar H, Yap VV. Recognition of useful music for emotion enhancement based on dimensional model. 2018. p. 176–80. | Scopus | Proceedings Paper | No |  |  | Wrong research type (conference) |
| Noor WMFWM, Zaini N, Norhazman H, Latip MFA. Dynamic encoding of binaural beats for brainwave entrainment. 2013. p. 626–30. | Scopus | Proceedings Paper | No |  |  | Wrong research type (conference) |
| Norhazman H, Mohamad Zaini N, Taib MN, Othman KA, Sani MM, Jailani R, et al. The effect of alpha binaural beat on frontal esd alpha asymmetry on different gender. ARPN Journal of engineering and applied sciences. 2016;11(7):4889–95. | Scopus | Journal Article | Yes | rpnjournals.org | No | Age unclear |
| Norhazman H, Zaini NM, Taib MN, Jailani R, Omar HA. The investigation of alpha frontal energy asymmetry on normal and stress subjects after listening to the binaural beats 10 Hz. 2014. p. 246–50. | Scopus | Proceedings Paper | No |  |  | Wrong research type (conference) |
| Norhazman H, Zaini NM, Taib MN, Omar HA, Jailani R, Lias S, et al. Behaviour of EEG alpha asymmetry when stress is induced and binaural beat is applied. 2012. p. 297–301. | Scopus | Proceedings Paper | No |  |  | Wrong research type (conference) |
| Norhazman H, Zaini N, Taib MN, Jailani R, Latip MFA. Alpha and beta sub-waves patterns when evoked by external stressor and entrained by binaural beats tone. 2019. p. 112–7. | Scopus | Proceedings Paper | No |  |  | Wrong research type (conference) |
| On FR, Jailani R, Norhazman H, Zaini NM. Binaural beat effect on brainwaves based on EEG. 2013. p. 339–43. | Scopus | Proceedings Paper | No |  |  | Wrong research type (conference) |
| Orozco Perez HD, Dumas G, Lehmann A. Binaural beats through the auditory pathway: From brainstem to connectivity patterns. eNeuro. 2020;7(2). doi: 10.1523/eneuro.0232-19.2020. | Scopus | Journal Article | Yes | ncbi.nlm.nih.gov | Yes |  |
| Ozdamar O, Bohorquez J, Mihajloski T, Yavuz E, Lachowska M. Auditory evoked responses to binaural beat illusion: stimulus generation and the derivation of the Binaural Interaction Component (BIC). Conference proceedings : Annual International conference of the IEEE engineering in medicine and biology society. 2011;2011:830–3. | Scopus | Proceedings Paper | No |  |  | Wrong research type (conference) |
| Park J, Kwon H, Kang S, Lee Y. The effect of binaural beat-based audiovisual stimulation on brain waves and concentration. 2018. p. 420–3. | Scopus | Proceedings Paper | No |  |  | Wrong research type (conference) |
| Pereda E, Ioannou C, Bhattacharya J. EEG functional brain connectivity changes associated to the perception of binaural beats: A study based on information theory. International journal of psychophysiology : official journal of the International Organization of Psychophysiology. 2014;94(2):194. doi: 10.1016/j.ijpsycho.2014.08.802. | WoS | Conference Lecture Abstract | No |  |  | Wrong research type (conference) |
| Phneah SW, Nisar H. EEG-based alpha neurofeedback training for mood enhancement. Australasian physical & engineering sciences in medicine. 2017;40(2):325–36. doi: 10.1007/s13246-017-0538-2. | Scopus | Journal Article | Yes | Springer Link | No | BBs embedded in music |
| Pratt H, Starr A, Michalewski HJ, Dimitrijevic A, Bleich N, Mittelman N. Cortical evoked potentials to an auditory illusion: Binaural beats. Clinical neurophysiology. 2009;120(8):1514–24. doi: 10.1016/j.clinph.2009.06.014. | Scopus | Journal Article | Yes | Elsevier | No | No bwe-hypothesis or measurement |
| Puzi NSM, Jailani R, Norhazman H, Zaini NM. Alpha and beta brainwave characteristics to binaural beat treatment. 2013. p. 344–8. | Scopus | Proceedings Paper | No |  |  | Wrong research type (conference) |
| Rahman JS, Gedeon T, Caldwell S, Jones RL. Can Binaural Beats increase your focus? Exploring the efects of music in participants' conscious and brain activity responses. Conference on human factors in computing systems - Proceedings; 2021. | Scopus | Proceedings Paper | No |  |  | Wrong research type (conference) |
| Ramdinmawii E, Mittal VK. The effect of music on the human mind: A study using brainwaves and binaural beats. 2018. p. 1–7. | Scopus | Proceedings Paper | No |  |  | Wrong research type (conference) |
| Rundshagen I, Kochs E, Bischoff P, Am Schulte Esch J. Intraoperative pain stimuli change somatosensory evoked potentials but not auditory evoked potentials during isoflurane/nitrous oxide anaesthesia. Anasthesiologie Intensivmedizin Notfallmedizin Schmerztherapie. 1997;32(10):604–9. doi: 10.1055/s-2007-995115. | Scopus | Journal Article | No |  |  | Wrong study goal |
| Schmid W, Marhofer P, Opfermann P, Zadrazil M, Kimberger O, Triffterer L, et al. Brainwave entrainment to minimise sedative drug doses in paediatric surgery: a randomised controlled trial. British journal of anaesthesia. 2020;125(3):330–5. doi: 10.1016/j.bja.2020.05.050. | Scopus | Journal Article | No |  |  | BBs combined with visual stimuli |
| Schwarz DWF, Taylor P. Human auditory steady state responses to binaural and monaural beats. Clinical neurophysiology : official journal of the International Federation of Clinical Neurophysiology. 2005;116(3):658–68. doi: 10.1016/j.clinph.2004.09.014. | Scopus | Journal Article | Yes | Elsevier | No | Participants under 18, no bwe-hypothesis |
| Seifi Ala T, Ahmadi-Pajouh MA, Nasrabadi AM. Cumulative effects of theta binaural beats on brain power and functional connectivity. Biomedical signal processing and control. 2018;42:242–52. doi: 10.1016/j.bspc.2018.01.022. | Scopus | Journal Article | Yes | Elsevier | Yes |  |
| Settapat S, Ohkura M. An alpha-activity-based binaural beat sound entrainment system using arousal state model. 2008. p. 63–6. | Scopus | Proceedings Paper | No |  |  | Wrong research type (conference) |
| Shamsi E, Ahmadi-Pajouh MA, Seifi Ala T. Higuchi fractal dimension: An efficient approach to detection of brain entrainment to theta binaural beats. Biomedical signal processing and control. 2021;68. doi: 10.1016/j.bspc.2021.102580. | Scopus | Journal Article | Yes | Elsevier | No | Same dataset as in Seifi Ala et al., 2018 (use as supporting data for discussion) |
| Sharma S, Rewadkar S, Pawar H, Deokar V, Lomte VM. Survey on binaural beats and background music for increased focus and relaxation. 2017. p. 98–103. | Scopus | Proceedings Paper | No |  |  | Wrong research type (conference) |
| Shumov DE, Arsen’ev GN, Sveshnikov DS, Dorokhov VB. Comparative analysis of the effect of stimulation with a binaural beat and similar kinds of sounds on the falling asleep process: A brief note. Moscow university biological sciences bulletin. 2017;72(1):33–6. doi: 10.3103/s0096392517010047. | Scopus | Journal Article | Yes | Springer Link | No | No bwe-measurement |
| Solcà M, Mottaz A, Guggisberg AG. Binaural beats increase interhemispheric alpha-band coherence between auditory cortices. Hearing research. 2016;332:233–7. doi: 10.1016/j.heares.2015.09.011. | WoS | Journal Article | Yes | sciencedirect | Yes |  |
| Stevens L, Haga Z, Queen B, Brady B, Adams D, Gilbert J, et al. Binaural beat induced theta EEG activity and hypnotic susceptibility: Contradictory results and technical considerations. The American journal of clinical hypnosis. 2003;45(4):295–309. doi: 10.1080/00029157.2003.10403543. | WoS | Journal Article | Yes | researchgate | Yes |  |
| Stone C, Thomas P, McClain-Furmanski D, Horton JE. EEG oscillations and binaural beat as compared with electromagnetic headphones and air-conduction headphones. Psychophysiology. 2002;39:S80-S. | WoS | Conference Poster | No |  |  | Wrong research type (conference) |
| Sujatha K, Kumaresan M, Ponmagal RS. Brain computer interface for vehicle automation. International journal of applied engineering research. 2014;9(24):29403–19. | Scopus | Journal Article | No |  |  | Wrong study goal |
| Ungan P, Yagcioglu S, Ayik E. Event-related potentials to single-cycle binaural beats and diotic amplitude modulation of a tone. Experimental brain research. 2019;237(8):1931–45. doi: 10.1007/s00221-019-05562-7. | Scopus | Journal Article | Yes | Springer Link | No | Secondary task, no no-bwe-comparison, no bwe-hypothesis |
| Ungan P, Yagcioglu S, Ayik E. Event-related potentials to single-cycle binaural beats of a pure tone, a click train, and a noise. Experimental brain research. 2019;237(11):2811–28. doi: 10.1007/s00221-019-05638-4. | Scopus | Journal Article | Yes | Springer Link | No | Secondary task, no no-bwe-comparison, no bwe-hypothesis |
| Vernon D, Peryer G, Louch J, Shaw M. Tracking EEG changes in response to alpha and beta binaural beats. International journal of psychophysiology : official journal of the International Organization of Psychophysiology. 2014;93(1):134–9. doi: 10.1016/j.ijpsycho.2012.10.008. | WoS | Journal Article | Yes | Elsevier | Yes |  |
| Vigil J, Tataryn L. Neurotherapies and Alzheimer's: A protocol-oriented review. NeuroRegulation. 2017;4(2):79–94. doi: 10.15540/nr.4.2.79. | Scopus | Journal Article | No |  |  | Wrong study goal |
| Wahbeh H, Calabrese C, Zwickey H, Zajdel D. Binaural beat technology in humans: A pilot study to assess neuropsychologic, physiologic, and electroencephalographic effects. Journal of alternative and complementary medicine (New York, NY). 2007;13(2):199–206. doi: 10.1089/acm.2006.6201. | WoS | Journal Article | Yes | researchgate | Yes |  |
| Woisard K, Stafford W, Klineberger P, Harrison K, Harrison D. JUST BEAT IT: BINAURAL BEATS IN THE BETA FREQUENCY. Psychophysiology. 2016;53:S60-S. | WoS | Conference Poster | No |  |  | Wrong research type (conference) |
| Yamsa-Ard T, Wongsawat Y. The observation of theta wave modulation on brain training by 5 Hz-binaural beat stimulation in seven days. 2015. p. 6667–70. | Scopus | Proceedings Paper | No |  |  | Wrong research type (conference) |
| Yamsa-Ard T, Wongsawat Y. The relationship between EEG and binaural beat stimulation in meditation. BMEiCON 2014 - 7th Biomedical engineering International conference; 2014. | Scopus | Proceedings Paper | No |  |  | Wrong research type (conference) |
| Yusim A, Grigaitis J. Efficacy of Binaural beat meditation technology for treating anxiety symptoms: A pilot study. Journal of nervous and mental disease. 2020;208(2):155–60. doi: 10.1097/nmd.0000000000001070. | Scopus | Journal Article | No |  |  | No EEG |
| Zaini N, Omar H, Latip MFA. Semantic-based Bayesian network to determine correlation between binaural-beats features and entrainment effects. 2011. p. 574–9. | Scopus | Proceedings Paper | No |  |  | Wrong research type (conference) |

**Citation Search**

| **Reference** | **Database** | **Research Type** | **Included at screening?** | **Obtained paper through** | **Included at selection?** | **Reason for exclusion** |
| --- | --- | --- | --- | --- | --- | --- |
| Pratt H, Starr A, Michalewski HJ, Dimitrijevic A, Bleich N, Mittelman N. A comparison of auditory evoked potentials to acoustic beats and to binaural beats. Hearing research. 2010;262(1-2):34–44. Epub 01.02.2010. doi: 10.1016/j.heares.2010.01.013. | / | Journal Article | Yes | sciencedirect | No | Secondary task |

**Search Rerun 2022**

| **Reference** | **Database** | **Research Type** | **Included at screening?** | **Obtained paper through** | **Included at selection?** | **Reason for exclusion** |
| --- | --- | --- | --- | --- | --- | --- |
| Baakek YNEH, Debbal SMEA. Digital drugs (binaural beats): how can it affect the brain/their impact on the brain. Journal of medical engineering and technology. 2021;45(7):546–51. doi: 10.1080/03091902.2021.1936236. | Scopus | Journal Article | No |  |  | Wrong study goal |
| Cheah KH, Nisar H, Tsai C-Y. Classification of music-induced mental states using convolutional neural networks for an EEG study. 2022. 383 p. | Scopus | Journal Article | No |  |  | Wrong study goal |
| Corona-González CE, Alonso-Valerdi LM, Ibarra-Zarate DI. Personalized theta and beta binaural beats for brain entrainment: An electroencephalographic analysis. Frontiers in psychology. 2021;12. doi: 10.3389/fpsyg.2021.764068. | Scopus | Journal Article | Yes | Frontiers in Psychology | Yes |  |
| Costa TDD, Godeiro C, Silva RAE, dos Santos SF, Machado DGD, Andrade SM. The Effects of non-invasive brain stimulation on quantitative EEG in patients with Parkinson's Disease: A systematic scoping review. Frontiers in neurology. 2022;13. doi: 10.3389/fneur.2022.758452. | WoS | Journal Article | No |  |  | Wrong research type (review) |
| Engelbregt H, Barmentlo M, Keeser D, Pogarell O, Deijen JB. Effects of binaural and monaural beat stimulation on attention and EEG. Experimental brain research. 2021;239(9):2781–91. doi: 10.1007/s00221-021-06155-z. | Scopus | Journal Article | No |  |  | Secondary task |
| Lee E, Bang Y, Yoon I-Y, Choi H-Y. Entrapment of binaural auditory beats in subjects with symptoms of insomnia. Brain sciences. 2022;12(3). doi: 10.3390/brainsci12030339. PubMed Central PMCID: PMC35326295. | Pubmed | Journal Article | No |  |  | Wrong sample (insomnia patients), EEG measured separately from BB stimulation |
| Mahmood D, Nisar H, Yap VV, Tsai C-Y. The effect of music listening on EEG functional connectivity of brain: A short-duration and long-duration study. MATHEMATICS. 2022;10(3). doi: 10.3390/math10030349. | Scopus | Journal Article | No |  |  | No passive comparison condition |
| Mujib MD, Hasan MA, Qazi SA, Vuckovic A. Understanding the neurological mechanism involved in enhanced memory recall task following binaural beat: a pilot study. Experimental brain research. 2021;239(9):2741–54. doi: 10.1007/s00221-021-06132-6. | WoS | Journal Article | Yes | Springer Link | No | Secondary task |
| Negi S, Jain R, Saboo S, Kwatra S. Automated generated binaural beats using EEG: A user-friendly auditory brain-computer interface. 2021 International conference on smart generation computing, communication and networking, SMART GENCON 2021; 2021. | Scopus | Proceedings Paper | No |  |  | Binaural beats mixed with neurofeedback methods |
| Rankhambe D, Ainapure B. EFFECT OF BINAURAL BEATS MUSIC THERAPY ON ANXIETY VIA EEG USING ANN & MACHINE LEARNING - A SURVEY. Journal of theoretical and applied information technology. 2022;100(3):630–47. | Scopus | Journal Article | No |  |  | No BWE measurement |
| Sadeghijam M, Talebian S, Mohsen S, Akbari M, Pourbakht A. Shannon entropy measures for EEG signals in tinnitus. Neuroscience letters. 2021;762. doi: 10.1016/j.neulet.2021.136153. | Scopus | Journal Article | No |  |  | Wrong sample (tinnitus patients) |
| Sadek RA, Khalifa AA, Elfattah MMA. Detecting digital stimulant music using bidirectional deep long short term memory. 2021. p. 150–4. | Scopus | Journal Article | No |  |  | Wrong study goal |
| Wang L, Zhang W, Li X, Yang S. The effect of 40 Hz binaural beats on working memory. IEEE Access. 2022:1. doi: 10.1109/access.2022.3185257. | Scopus | Journal Article | No |  |  | Secondary task |

**Search Rerun 2023**

| **Reference** | **Database** | **Research Type** | **Included at screening?** | **Obtained paper through** | **Included at selection?** | **Reason for exclusion** |
| --- | --- | --- | --- | --- | --- | --- |
| Alonso-Valerdi LM, Ibarra-Zárate DI, Torres-Torres AS, Zolezzi DM, Naal-Ruiz NE, Argüello-García J. Comparative analysis of acoustic therapies for tinnitus treatment based on auditory event-related potentials. Front Neurosci. 2023; 17:1059096. doi: 10.3389/fnins.2023.1059096 PMID: 37081936. | Pubmed | Journal Article | No |  |  | Wrong study goal |
| Alonso-Valerdi LM, Torres-Torres AS, Corona-González CE, Ibarra-Zárate DI. Clustering approach based on psychometrics and auditory event-related potentials to evaluate acoustic therapy effects. BIOMEDICAL SIGNAL PROCESSING AND CONTROL. 2022; 76. doi: 10.1016/j.bspc.2022.103719. | Scopus | Journal Article | No |  |  | Wrong study goal |
| Borges LR, Arantes A, Naves E. Influence of Binaural Beats Stimulation of Gamma Frequency over Memory Performance and EEG Spectral Density. Healthcare (Switzerland). 2023; 11. doi: 10.3390/healthcare11060801. | Scopus | Journal Article | Yes | MDPI | No | BBs embedded in music |
| Chernetchenko D, Prasolov P, Aganov S, Voropai A, Polishchuk Y, Lituiev D, et al. Effects of Binaural Beat Stimulation in Adults with Stuttering. Brain Sci. 2023; 13. doi: 10.3390/brainsci13020309. | Scopus | Journal Article | Yes | MDPI | No | BBs embedded in music, secondary task |
| Choi M-H, Jung J-J, Kim K-B, Kim Y-J, Lee J-H, Kim H-S, et al. Effect of binaural beat in the inaudible band on EEG (STROBE). Medicine (United States). 2022; 101:1–4. doi: 10.1097/MD.0000000000029819. | Pubmed | Journal Article | No |  |  | Carrier tones in the inaudible auditory range |
| Cuevas-Romero AR, Alonso-Valerdi LM, La Intriago-Campos, Di Ibarra-Zarate. An Electroencephalography-based Database for studying the Effects of Acoustic Therapies for Tinnitus Treatment. Sci Data. 2022; 9. doi: 10.1038/s41597-022-01622-w. | WoS | Journal Article | No |  |  | Wrong study goal |
| Gantt MA. Study protocol to support the development of an all-night binaural beat frequency audio program to entrain sleep. Frontiers in Neurology. 2023; 14. doi: 10.3389/fneur.2023.1024726. | Scopus | Journal Article | No |  |  | Wrong study sample (patients with sleep problems) |
| González D, Bruña R, Martínez-Castrillo JC, López JM, Arcas G de. First Longitudinal Study Using Binaural Beats on Parkinson Disease. Int J Neural Syst. 2023:2350027. doi: 10.1142/S0129065723500272 PMID: 37085963. | Pubmed | Journal Article | No |  |  | Wrong study sample (Parkinson’s patients) |
| Katmah R, Al-Shargie F, Tariq U, Babiloni F, Al-Mughairbi F, Al-Nashash H. Mental Stress Management Using fNIRS Directed Connectivity and Audio Stimulation. IEEE TRANSACTIONS ON NEURAL SYSTEMS AND REHABILITATION ENGINEERING. 2023; 31:1086–96. doi: 10.1109/TNSRE.2023.3239913. | Scopus | Journal Article | No |  |  | No BWE measurement |
| Kim K-B, Jung J-J, Lee J-H, Kim Y-J, Kim J-S, Choi M-H, et al. Frequency-following response effect according to gender using a 10-Hz binaural beat stimulation. Technol Health Care. 2023. doi: 10.3233/THC-236001 PMID: 37038776. | Pubmed | Journal Article | Yes | IOS Press | Yes |  |
| Kweon Y-S, Shin G-H, Kwak H-G, editors. Development of Personalized Sleep Induction System based on Mental States. ; 2023. | Scopus | Proceedings Paper | No |  |  | Wrong research type |
| Lee M, Lee HJ, Ahn J, Hong JK, Yoon IY. Comparison of autonomous sensory meridian response and binaural auditory beats effects on stress reduction: a pilot study. Sci Rep. 2022; 12. doi: 10.1038/s41598-022-24120-w. | WoS | Journal Article | No |  |  | Wrong study sample (patients with stress) |
| Liu Z-B, Liu Y-S, Zhao L, Li M-Y, Liu C-H, Zhang C-X, et al. Short-term efficacy of music therapy combined with α binaural beat therapy in disorders of consciousness. Front Psychol. 2022; 13. doi: 10.3389/fpsyg.2022.947861. | Scopus | Journal Article | No |  |  | Wrong study sample (patients with disorders of consciousness) |
| Thanyawinichkul K, Tontisirin N, Mahawan R, Kumdang S, Yamsa-Ard T, Maneepairoj M, et al. The Efficacy of Binaural Beat Stimulation Mixed with Acoustic Music in Chronic Low Back Pain Management: A Randomized Controlled Trial. Journal of the Medical Association of Thailand. 2022; 105:806–14.doi: 10.35755/jmedassocthai.2022.09.13598. | Scopus | Journal Article | No |  |  | BBs embedded in music |
| Wang X, Lu H, He Y, Sun K, Feng T, Zhu X. Listening to 15 Hz Binaural Beats Enhances the Connectivity of Functional Brain Networks in the Mental Fatigue State—An EEG Study. Brain Sci. 2022; 12. doi: 10.3390/brainsci12091161. | Scopus | Journal Article | No |  |  | Secondary task |
| Yi J-H, Kim K-B, Kim Y-J, Kim J-S, Kim H-S, Choi M-H, et al. A Comparison of the Effects of Binaural Beats of Audible and Inaudible Frequencies on Brainwaves. Applied Sciences (Switzerland). 2022; 12. doi: 10.3390/app122413004. | Scopus | Journal Article | Yes | MDPI | No | Same dataset as in Kim et al. (2023) |
